# Detection of Common Causes between Air Traffic Serious and Major Incidents in Applying the Convolution Operator to Heinrich Pyramid Theory

**DOI:** 10.3390/e21121166

**Published:** 2019-11-28

**Authors:** Schon Z. Y. Liang Cheng, Rosa Maria Arnaldo Valdés, Víctor Fernando Gómez Comendador, Francisco Javier Sáez Nieto

**Affiliations:** 1 Department of Sistemas Aeroespaciales, Transporte Aéreo y Aeropuertos, School of Aerospace Engineering, Universidad Politécnica de Madrid (UPM), Plaza Cardenal Cisneros n3., 28040 Madrid, Spain; fernando.gcomendador@upm.es; 2 Aeronautic, Space & Defence Division, ALTRAN Innovation S.L., Calle Campezo 1, 28022 Madrid, Spain; 3 Centre for Aeronautics, School of Aerospace, Transport and Manufacturing, Cranfield University, Cranfield, Bedford MK43 0AL, UK; p.saeznieto@cranfield.ac.uk

**Keywords:** Heinrich’s pyramid theory, convolutional matrix, ATM incident analysis, information theory, aviation safety

## Abstract

Heinrich’s pyramid theory is one of the most influential theories in accident and incident prevention, especially for industries with high safety requirements. Originally, this theory established a quantitative correlation between major injury accidents, minor injury accidents and no-injury accidents. Nowadays, researchers from different fields of engineering also apply this theory in establishing quantitatively the correlation between accidents and incidents. In this work, on the one hand, we have detected the applicability of this theory by studying incident reports of different severities occurred in air traffic management. On the other hand, we have deepened the analysis of this theory from a qualitative perspective. For this purpose, we have applied the convolution operator in identifying correlations between contributing causes to different incident severities, also known as precursors to accidents, and system failures. The results suggested that system failures are mechanisms by which the causes are manifested. In particular, the same underlying cause can be manifested through different failures which contribute to incidents with different severities. Finally, deriving from this result, an artificial neuronal network model is proposed to recognize future causes and their possible associated incident severities.

## 1. Introduction

Heinrich’s Pyramid Theory is another influential theory such as the Swiss chess model (SCM) of Reason [1] in safety science. This theory suggests that minimizing the number of incidents with lower levels of severities leads to reducing the number of high severity events, including accidents [2]. According to this theory, a large number of incidents with low consequences, if untreated, would potentially lead to few occurrences with high consequences [3]. Moreover, a progressive increase in minor incidents would lead to a major accident. Whilst some researchers disagree with Heinrich by stating that accidents are caused by poor management systems as the main reason and not by human actions [4] and provide criticism related to the lack of qualitative representation of this theory [5], the pyramid theory is still widely applied for safety management in different sectors. Kyriakidis et al. [6] have deepened this theory in improving accident precursor monitoring program of railway safety; Golovina et al. [7] have designed an algorithm based on this theory for preventative hazard recognition and control process related to construction safety. Marshall et al. [8] have turned to statistical methods to confirm Heinrich’s theory in occupational accidents. For industrial process analysis, Prem et al. [9] have generated safety pyramids based on historical databases of chemical industrial accidents and compared them with Heinrich’s pyramid to understand incident occurrence trends.

Particularly, in the aviation sector, Walker [10] has established a risk pyramid with quantitative relation between occurrences, incidents, and accidents based on data registered in black boxes with the purpose of improving flight data monitoring system. Majumdar et al. [11] have applied this theory directly to develop safety indicators using the data of loss of separation (LOS) incidents registered in airspaces of New Zealand and the United Kingdom; however, unlike the quantitative relation considered in Heinrich’s pyramid, Nazeri and Lance [12] have applied this theory in looking for a qualitative relation between accidents and incidents through their underlying factors. 

Most of these researchers have used big data sources to demonstrate the validity of Heinrich’s pyramid theory [8], and thus show the proportion between occurrences with different levels of severity [12]. Based on this theory, they have established a quantitative relationship between occurrences with their source data. Even Heinrich in [2] postulated that, for each accident with major injury, there were 29 accidents with minor injuries and 300 accidents without injuries. However, both Heinrich and these researchers have not examined the mode of connection or contribution of underlying causes to occurrences with different levels of severity. Such a qualitative relationship is no less important than the quantitative one and it might support us in understanding the stream of causes from a low to a high level of severity.

From this perspective, statistical models that can establish the qualitative relationship between different levels of the pyramid will be advantageous in comprehending the proximity to fatalities [9]. In our previous work [13], we followed a series of steps in extracting serious incident data for Bayesian Network (BN) construction as well as searching possible scenarios where influential causes contributed to this category of accidents. In our research [14], we have completed the analysis adding major incidents and updated the BN model providing relations between serious (near accidents) and major incidents, which have been established through the connections between factors and events in different categories of the incident. Thus, one qualitative study related to the connections should be necessary and support us to detect the behaviour of each factor in different categories of incidents, even its associated events. For this reason, we employed the use of convolution operator, one mathematical operator, in filtering [15] and amplifying the information [16] contained in this kind of factors. 

### Objectives

In our previous work [14], our results indicated that some causes contribute to different categories of incidents. Their combinations provide potential scenarios leading to an accident in one category of incidents, but not in another. Derivate from this result, we can observe that common factors can be identified connecting different categories of incidents with different contributions. Therefore, the analysis should be deepened in the following points:

- Apply Heinrich’s Pyramid Theory in studying air traffic management (ATM) incidents. Based on the results in [13,14], it is deduced that a relationship might be established between factors and categories of incidents; such relationship approximates that described by Heinrich’s Pyramid Theory concerning causes and levels of severity. In addition, to check whether this theory explains the results obtained in previous papers, we are also interested in knowing if one relationship would be established between causes and different categories of incidents occurring within the ATM system.
- Detect correlations between factors and different levels of incident severity. If factors connect between different levels, we need to know what correlation is established between factors and incident severity levels. In this manner, it is possible to study the behaviour of each factor and its mode of contribution or stream within these incidents.

## 2. Material 

According to ICAO Annex 11 [17] and European Regulation (EU) No. 376/2014 [18], an incident investigation must be conducted by the local authority and its final report should be published. In Spain, the State Investigation Office has the responsibility of receiving the notification and proceeding with the corresponding investigation. This entity is also in charge of processing the incident data and publishing the final report [19]. Table 1 presents a set of occurrences and categories of all investigated incidents, which occurred in the Spanish ATM system during four consecutive years. Within 31 serious incidents (severity A) and 139 major incidents (severity B), near 50% of them correspond to LOS incidents occurring between aircraft. Focusing on the purpose of this research work, only LOS incidents between commercial aircraft have been considered, resulting in a sample of 87 LOS incident reports in total; 14 serious incidents and 73 major incidents have been analysed. 

## 3. Methodology 

**Figure 1:**
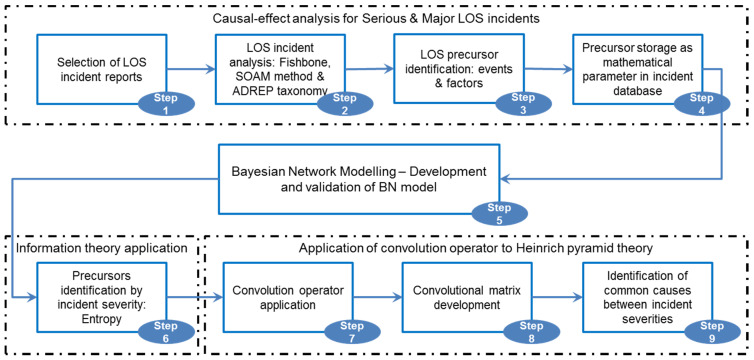
Methodology of convolution operator application to Heinrich pyramid theory in detecting common causes between incident severities.

Steps of the methodology that we have followed during this research work are indicated in Figure 1. Even though steps 1–6 have been already exhaustively defined in our previous publications [13,14], they are summarized below in keeping the contextual connection. 

The initial phase (steps 1–4) aims to detect causes and failures contributing to LOS serious and major incidents. Data collected from these incident reports are identified as factors and events, which are also denominated as precursors to future accidents. These factors and events can be extracted and codified by standardized methodologies [20,21,22] and taxonomies [23], which have been applied in this process. Factors based on taxonomy can be divided into two groups: descriptive factor (DF) and explanatory factor (EF). Both groups of factors represent causes of failures, meanwhile, events are identified as failures of the system.

In the second phase (step 5) based on the established correlation between factors and events, a BN model can be developed and validated. Moreover, a quantitative cause–effect map can be depicted through the BN model (factors as children nodes and events as parent nodes) and used to recreate scenarios of serious and major LOS incidents. Within the BN model, the likelihoods of factors and events, as well as their strength of the connections, are estimated based on the number of analysed incident reports and collected in the conditional probability table (CPT) [24].

During the third phase (step 6) the information theory developed from entropy principals is applied to identify the most correlated precursors of serious and major LOS incidents. The mutual information concept is used in quantifying the contribution of causes to these two incident severities and formulated as Equation (1):

(1) $$I\left(Z,Y\right)=H\left(Z\right)-H\left(Z|Y\right)=\underset{z,y}{\sum }P\left(z,y\right)log\frac{P\left(z,y\right)}{P\left(z\right)P\left(y\right)}=\underset{z,y}{\sum }P\left(y\right)P\left(z|y\right)log\frac{P\left(z|y\right)}{P\left(z\right)}$$

During the last phase, Heinrich’s Pyramid Theory is considered in analysing precursors. Regarding Heinrich’s Pyramid Theory, factors that contribute to critical incidents, or with a higher severity level, are also present in less critical incidents or lower levels of severity. The application of this theory affords the identification of factors that have been involved in the incidents of severity A and B, and reveal their modes of participation in the incidents. However, this theory provides less qualitative correlation, which indicates the mode of contributing and the connection of these factors within two proximate severities. Consequently, without knowing the detail of this correlation, suitable design of barriers that allow the effective mitigation of events would not be carried out. Therefore, we can deepen the analysis by identifying the factors that chain between severity levels (concatenated factors), and their connectivity behaviours within different categories of incidents caused by them. Hence, Equation (2) of convolution for discrete sets [25] is applied to two sets of incidents with different severities, thereby filtering and amplifying information on factors common to both categories of incidents (step 7). 

Equation (2):

(2) $$I{\left[k\right]}_{f}*I{\left[k\right]}_{g}=\underset{l}{\sum }I{\left[l\right]}_{f}I{\left[k-l\right]}_{g}$$

where *I_f_* and *I_g_* are functions of mutual information of two sets of incidents with different and proximate severities. Additionally, according to the commutative property of convolution, $I_{f}*I_{g}=I_{g}*I_{f}$, the convolution from one set to another presents a symmetrical interpretation. Developing Equation (2), one generic convolution matrix related to the status of mutual information of a factor in two close severities is created as indicated in Table 2 (step 8). 

Three associated situations of incidents are shown independently of the factor states (columns of the matrix):

i. Both categories of incidents are in present states;
ii. One of them is in the present state and the other one in the absent state;
iii. Neither of them is in the present state.

The other three situations associated with the status of the factor are shown independently of the incident states (rows of the matrix):

i. The factor belongs to both incident categories;
ii. The factor only belongs to one of both incident categories;
iii. The factor is from neither of both incident categories.

Finally, the total number of mutual information that both severities of incidents share by this factor is the sum (*I*) of these nine components in the convolution matrix. Depending on the result of this sum of mutual information, three cases related to the participation and the behaviour of factors in different incident severities can be discussed (step 9).

## 4. Results of Application

As input data, a set of serious and major LOS incidents occurred between commercial aircraft in the Spanish airspace during four consecutive years has been considered (step 1). The analysis of incident reports provides causal–effect paths leading to serious and major LOS (step 2), and precursors that are extracted and attributed to events and factors (step 3). For the purpose of data management, these precursors are registered in a database as mathematical parameters (step 4).

**Figure 2:**
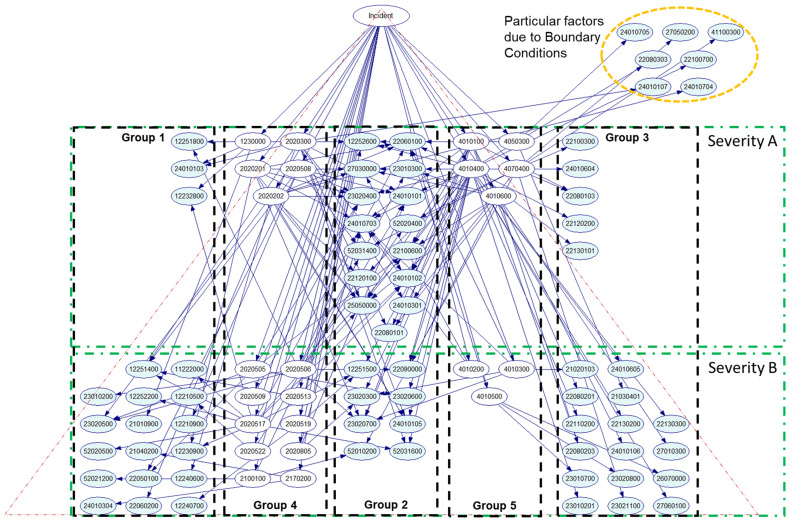
Heinrich’s pyramid Bayesian Network (BN) model for loss of separation (LOS) serious and major incidents in commercial aviation.

Figure 2 illustrates the proposed BN model in this research work. The model is a transformation from the result published in [14] with Heinrich’s pyramid theory in consideration (step 5). The CPT of correlation between factors and events is the same as published in [14] and summarized in Appendix A. In addition, accident/incident data reporting (ADREP) codifications of events and descriptive factors implicated in this research work are listed in Appendix B.

Events and factors have been divided into five groups within this BN model:

- Group 1, children nodes on the air side, group of DFs related to A/C or flight crew.
- Group 2, children nodes of connection, group of DFs related to A/C or flight crew—ATM.
- Group 3, children nodes on the ground side, groups of DFs related to ATM.
- Group 4, parent nodes on the air side, group of events related to A/C or flight crew.
- Group 5, parent nodes on the ground side, group of events related to ATM.

The difference with respect to results represented in [14] is that, after considering Heinrich’s pyramid theory, events and factors can be organized and presented in such manner that they are associated with different levels of severity. In other words, with Figure 2, events and factors in severity A level are common to both incident severities. Meanwhile, events and factors in severity B level are singular from major incidents. 

From the BN model, the likelihood of each factor is used to estimate its mutual information. Applying Equation (1), we obtain two matrices of mutual information of LOS incidents with severity A and B, ${ℳ\left(I\right)|}_{A}$ and ${ℳ\left(I\right)|}_{B}$ (step 6) and the sum of their components in each matrix is the mutual information for a particular DF in our validated BN model, $I_{A}\left({DF}_{i}\right)$ and $I_{B}\left({DF}_{i}\right)$. Applying Equation to both matrices: ${ℳ\left(I\right)|}_{A}⨂{ℳ\left(I\right)|}_{B}={ℳ\left(I\right)|}_{B}⨂{ℳ\left(I\right)|}_{A}$ (step 7). Then the convolution matrix of each DF is calculated and shown in Table 3 (step 8).

Moreover, the sum of its components, ${I\left(Z,Y\right)|}_{A\cap B}=\underset{z,y}{\sum }{I\left(i,j\right)|}_{A\cap B}$, is the mutual information of each DF in both severity A and B (I_A∩B). 

As a result, we have three vectors of mutual information for all DFs contributed in the validated BN model: $I_{A}\left(DF_{1},DF_{2},\cdots ,DF_{n}\right)$, $I_{B}\left(DF_{1},DF_{2},\cdots ,DF_{n}\right)$ and $I_{A\cap B}\left(DF_{1},DF_{2},\cdots ,DF_{n}\right)$. For facilitating the analysis, each vector is normalized with respect to the sum of all its components. 

**Figure 3:**
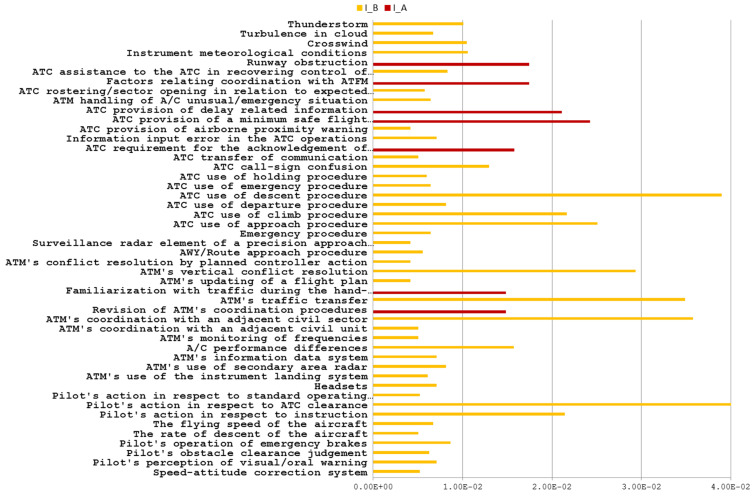
Descriptive factors (DFs) with I_A∩B = 0.

Regarding the estimated mutual information that measures the participation of common factors in both categories of incidents, the factors can be identified within the following three groups (step 9): 

Group 9.1. As shown in Figure 3, all factors have I_A∩B = 0. It means that no mutual information is shared between both severities by the same factor, and these kinds of factors with such characteristics are listed in Table A5 of Appendix C and belong to one category of incidents only. According to Heinrich’s pyramid theory, these kinds of factors should be specific to incidents with low severity level, i.e., severity B in this case. 

**Figure 4:**
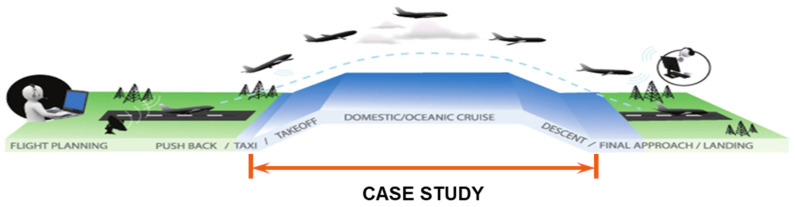
Boundary conditions of the case study.

However, there are ones listed in Table A6 that belong to incidents of severity A, the high severity level. This singularity exists when the study is limited by the established boundary conditions for our case study:

- Incident severity: serious and major incidents are considered;
- Incident category: LOS or separation minima infringement (SMI);
- Type of flight: limited only to commercial aircraft involved in the incident scenario;
- Operating phase: none of the involved aircraft were operating at the final approach phase or before achieving the second segment of the take-off, as indicated in Figure 4.

If these boundary conditions are removed, i.e., extending cases studies considering other types of flight like incidents occurred between military and civil aircraft, these factors would be present in incidents of severity B, lower level of severity, regarding Heinrich’s Pyramid Theory.

**Figure 5:**
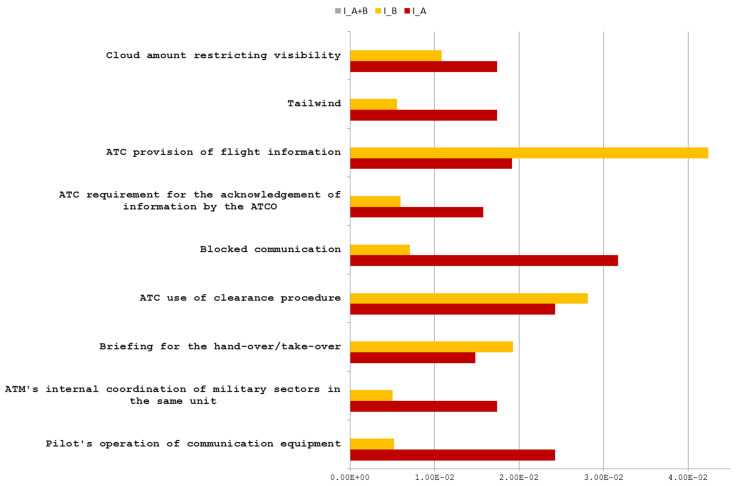
DFs with 1 × 10^−10^ < I_A∩B < 1 × 10^−3^(I → 0).

Group 9.2. As shown in Figure 5, all factors listed in Table A7 have I_A∩B → 0. The mutual information shared by factors within all incidents of severity A and B are close to zero. It means that these factors provide a weak connection to both severity levels. 

According to the property of Kullback-Leibler divergence (KLD) [26,27], these factors contribute to both severities separately. In other words, they are in present either in severity A incidents or in severity B incidents. This result, checked together with the BN model, shows that all factors are linked to two independent joints of events, such that each joint belongs to one specific category of incidents without intersection with others. For example, if the factor ‘24010103 Blocked communication’ is in the present state, then events ‘2020300 Communication between pilot and ANS’ and ‘1230000 Communication systems’ could be affected. However, the event ‘2020300 Communication between pilot and ANS belongs to severity A incidents, meanwhile the event ‘1230000 Communication systems’ belongs to severity B incidents only.

**Figure 6:**
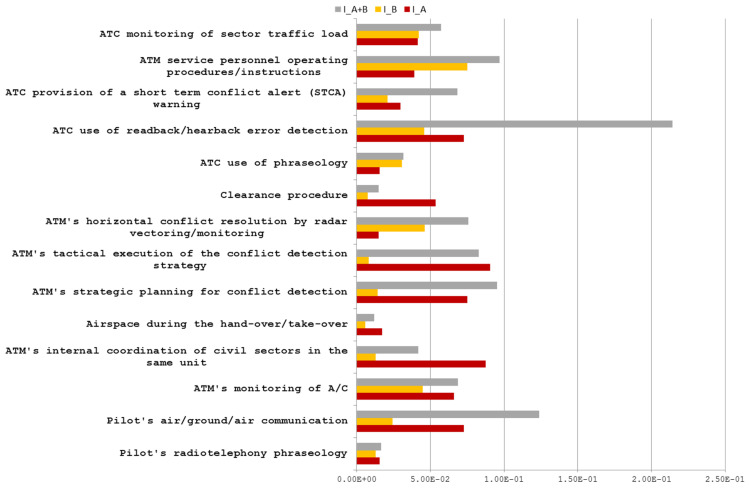
DFs with I_A∩B > 1 × 10^−3^.

Group 9.3. As shown in Figure 6 all factors listed in Table A8 contribute to both categories of incidents through common events. 

These events leading to either of the two incident categories are manifested, whilst the factors are in the present state. For example, when the factor ‘24010102 ATC use of readback/hearback error detection’ is in the present state, then the events in Table 4 could be affected. Moreover, the mutual information of this factor is higher than others due to its stronger connection to both severities through the event ‘2020300 Communication between pilot and ANS’.

**Figure 7:**
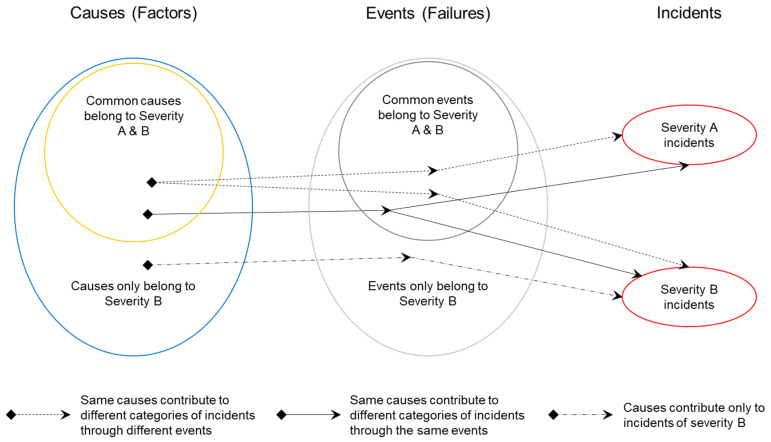
Contribution path of causes to incidents.

In summary, contribution paths of causes to incidents are performed through events in three paths as indicated in Figure 7:

i. Causes only belong to severity B incidents contribute exclusively to this category of incidents, then the mutual information of both categories of incidents is zero (I_A∩B = 0);
ii. Common causes belong to incidents of severity A and B can contribute to each category of incidents through the same mechanisms or events. In this case, the mutual information of both categories of incidents is different to zero (I_A∩B ≠ 0);
iii. Common causes belong to incidents of severity A and B and contribute to different categories of incidents through different mechanisms or events. In this case, the mutual information of both categories of incidents tends to zero (I_A∩B → 0).

## 5. ANN Model Proposal from the Analysis Result

**Figure 8:**
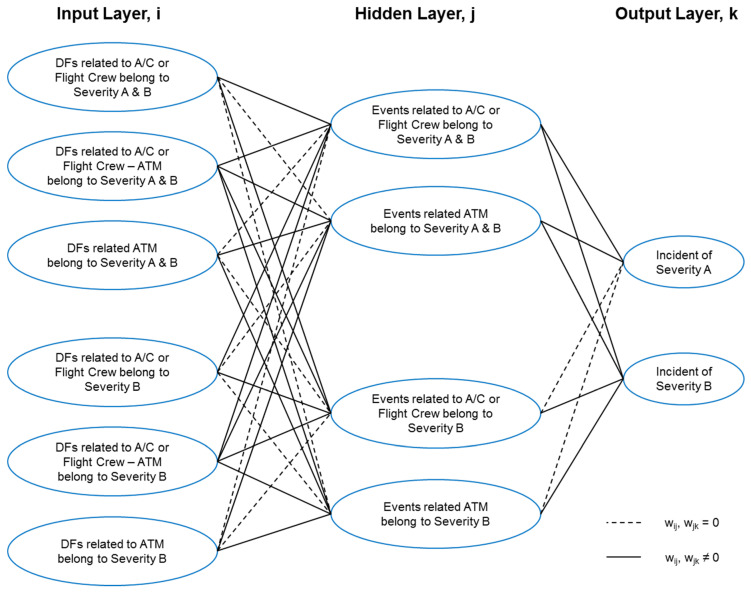
Convolutional neuronal network.

In addition, based on the analysis results and this reorganization of the BN model, connections between different groups of events and factors provide other interpretations with a tendency to possible applications of neuronal networks. As indicated in Figure 8, this simple multilayer Perceptrons (MLP) neuronal network consists of three layers:

- Input layer, *i*: shaped by classified groups of factors (*x_i_*);
- Hidden layer, *j*: performed by events groups (*y_j_*);
- Output layer, *k*: provided by results of incident prediction (*O_k_*).

Therefore, the general MLP equation for each layer can be formulated as follows:

Equation (3):

(3) $$x_{i}=P\left(DF_{i}\right)$$

Equation (4):

(4) $$y_{j}=\sum w_{ij}I_{i}+b_{j},\;\mathrm{being}\;I_{i}=f\left(x_{i}\right)$$

Equation (5):

(5) $$O_{k}=\sum w_{jk}y_{j}+b_{k}$$

In Equation (4) and Equation (5), *w_ij_* and *w_jk_* are weight parameters after the convolution for estimated mutual information, depending on the participation of the DF in different incident categories, i.e., if one DF belongs to severity A incident only, then the *w_ij_* for events of severity B incidents are null (*w_ij_* = 0); meanwhile *x_i_* (input layer) is the estimated likelihood of each DF in the BN model, *y_j_* (hidden layer) and *O_k_* (output layer) correspond to the mutual information in the function of *x_i_*. Note that *bi* and *bj* are bias for additional weight adjustments in neuronal networks.

The method of applying Bayesian network to neuronal networks training became popular, researchers like Huang et al. [28] applied this method for foreign exchange rates forecasting, Abdulhai et al. [29] used it for freeway incident detection and Gupta and Schumann [30] implemented it for improving flight control system.

Unlike other researchers that have used Bayesian network as a data filter for neuronal network training, through this analysis we attempt to show a possible construction of a Bayesian-driven neuronal network model. In this manner, we could have a neural network with its hidden layer controlled. When a factor is located in a possible occurrence, we would know with which event group this factor would be associated and to which incident category it would be contributed.

## 6. Conclusions

In this analysis, Heinrich’s Pyramid Theory has been considered as the main approach that allowed the detection of common factors within different levels of severity as well as their relationship. According to this theory, causes detected at high levels of severity are always found at low levels; therefore, these causes are identified as concatenated factors, which contribute to incidents through their pertinent events. 

Moreover, we have explored this theory in depth through the analysis of mutual information between both severity levels, and introduced it in refining the contribution of factors to different categories of incidents.

For deepening the analysis, we have selected all LOS incidents of severity A and B that occurred in the Spanish airspace during four consecutive years. The selection of these two joints of incidents has been specified by defined boundary conditions. The equation of convolution for discrete sets is applied in estimating qualitatively the mutual information between these incident joints, and hence the behaviour of factors and their modes of contribution within incidents depending on values of mutual information. 

### 6.1. Benefits of the Application

The application of this methodology illustrates how the simple application of the convolution operator to Heinrich’s pyramid theory makes clearer the contribution of causes in incidents occurred due to operational failures. The added value of this technique allows us to detect contributing paths of causes leading to incidents. 

Additionally, with the filtration of mutual information calculated within different incident severities, the correlation between causes, failures and incident categories are identified more clearly. We can observe that some common factors (causes) provide common events (failures) and belong to both incident severities. However, from these events, different paths have been separated into two categories of incidents, i.e., with determinate factors, some events only contribute to severity A or B incidents and others contribute to both categories of incidents. In other words, the same causes detected in different categories of incidents can provide different streams through various failures. Consequently, although we know the causes of operational failures, one solution focused on avoiding the failures does not prevent incidents occurring. Indeed, this conclusion could guide us to reassess the design of barriers in avoiding the recurrence of causes. 

### 6.2. Limitations of the Application

The proposed methodology presents limitations as follows:

- Computational limitation: Although one neuronal network model based on the BN approach can be proposed, the number of cases for network learning is limited due to serious and major incidents occurring rarely.
- Data limitation: Causes and failures of serious and major incidents are known only from incident reports, or their frequencies of occurrence are partially known. Therefore, data related to their contributions to non-incident operations or incidents with less severity, i.e., minor incidents (severity C), are missed and, consequently, the accuracy of the information theory approach is compromised due to data limitations.
- BN model limitation: the model requires continuous updating of data to provide a higher level of reliability and reduce the degree of uncertainty.

### 6.3. Future Work

- The proposed Bayesian-driven neuronal network model is limited to a conceptual design currently. Thus, more cases of serious and major incidents should be analysed and used for model learning.
- Regarding the computational limitation, minor incidents could be considered to complete the correlation between causes and failures. It might be interesting to check the behaviour of already established contribution paths with this new severity level in consideration.

## Figures and Tables

**Table 1:** Spanish incident reports during four consecutive years.

Incident Category	N° Incidents—Year 1	N° Incidents—Year 2	N° Incidents—Year 3	N° Incidents—Year 4	N° Incidents—Total
A	13	5	10	3	31
B	37	31	37	34	139
C	40	38	53	50	181
D	2	1	1	0	4
E	2	1	5	3	11
TOTAL	94	76	106	90	366

**Table 2:** Mutual information matrix for two proximate categories of incidents.

${I\left(1,1\right)|}_{f}\cdot {I\left(1,1\right)|}_{g}$	${I\left(1,1\right)|}_{f}\cdot {I\left(1,2\right)|}_{g}$ $+{I\left(1,2\right)|}_{f}\cdot {I\left(1,1\right)|}_{g}$	${I\left(1,2\right)|}_{f}\cdot {I\left(1,2\right)|}_{g}$
${I\left(1,1\right)|}_{f}\cdot {I\left(2,1\right)|}_{g}$ $+{I\left(2,1\right)|}_{f}\cdot {I\left(1,1\right)|}_{g}$	${I\left(1,1\right)|}_{f}\cdot {I\left(2,2\right)|}_{g}+{I\left(1,2\right)|}_{f}\cdot {I\left(2,1\right)|}_{g}$ $+{I\left(2,1\right)|}_{f}\cdot {I\left(1,2\right)|}_{g}+{I\left(2,2\right)|}_{f}\cdot {I\left(1,1\right)|}_{g}$	${I\left(1,2\right)|}_{f}\cdot {I\left(2,2\right)|}_{g}$ $+{I\left(2,2\right)|}_{f}\cdot {I\left(1,2\right)|}_{g}$
${I\left(2,1\right)|}_{f}\cdot {I\left(2,1\right)|}_{g}$	${I\left(2,1\right)|}_{f}\cdot {I\left(2,2\right)|}_{g}$ $+{I\left(2,2\right)|}_{f}\cdot {I\left(2,1\right)|}_{g}$	${I\left(2,2\right)|}_{f}\cdot {I\left(2,2\right)|}_{g}$

**Table 3:** Mutual information matrix for incidents of severity A and B.

${I\left(1,1\right)|}_{A}\cdot {I\left(1,1\right)|}_{B}$	${I\left(1,1\right)|}_{A}\cdot {I\left(1,2\right)|}_{B}$ $+{I\left(1,2\right)|}_{A}\cdot {I\left(1,1\right)|}_{B}$	${I\left(1,2\right)|}_{A}\cdot {I\left(1,2\right)|}_{B}$
${I\left(1,1\right)|}_{A}\cdot {I\left(2,1\right)|}_{B}$ $+{I\left(2,1\right)|}_{A}\cdot {I\left(1,1\right)|}_{B}$	${I\left(1,1\right)|}_{A}\cdot {I\left(2,2\right)|}_{B}+{I\left(1,2\right)|}_{A}\cdot {I\left(2,1\right)|}_{B}$ $+{I\left(2,1\right)|}_{A}\cdot {I\left(1,2\right)|}_{B}+{I\left(2,2\right)|}_{A}\cdot {I\left(1,1\right)|}_{B}$	${I\left(1,2\right)|}_{A}\cdot {I\left(2,2\right)|}_{B}$ $+{I\left(2,2\right)|}_{A}\cdot {I\left(1,2\right)|}_{B}$
${I\left(2,1\right)|}_{A}\cdot {I\left(2,1\right)|}_{B}$	${I\left(2,1\right)|}_{A}\cdot {I\left(2,2\right)|}_{B}$ $+{I\left(2,2\right)|}_{A}\cdot {I\left(2,1\right)|}_{B}$	${I\left(2,2\right)|}_{A}\cdot {I\left(2,2\right)|}_{B}$

**Table 4:** Associated events in severity A and B when DF 24010102 is in the present state.

Severity	Event ID	Events Associated to DF 24010102
A&B	2020300	Communication between pilot and ANS
B	2020513	Clearance deviation - special procedure
B	2020517	Deviation from clearance - assigned flight level
A	4010100	ANS operational communications
B	4010400	ANS conflict detection and resolution

## Abbreviations

**ABBREVIATION**	**DEFINITION**
A/C	Aircraft
ADREP	Accident/Incident Data Reporting
ANS	Air Navigation Service
ATC	Air Traffic Control
ATCO	Air Traffic Control Officer
ATM	Air Traffic Management
BN	Bayesian Network
CPT	Conditional Probability Table
DF	Descriptive Factor
EF	Explanatory Factor
ICAO	International Civil Aviation Organization
KLD	Kullback-Leibler divergence
LOS	Loss of Separation
MLP	Multilayer Perceptrons
SCM	Swiss Chess Method
SMI	Separation Minima Infringement
STCA	Short Term Conflict Alert

## Appendix A. CPT of Events and Factors for BN Modelling

**Table A1:** CPT of events and descriptive factors under scenario of LOS in commercial aviation—Severity A.

Adverse Events (E)	Event Definition	P(E)	P(E|Severity)	Descriptive Factors (DF)	Descriptive Factor Definition	P(DF)	P(DF|E)
1230000	Communication systems	1.15 × 10^−2^	7.14 × 10^−2^	12232800	Pilot’s operation of communication equipment	1.15 × 10^−2^	5.00 × 10^−1^
2020201	ANS erroneous clearance	3.45 × 10^−2^	2.14 × 10^−1^	22060100	ATM’s monitoring of A/C	1.15 × 10^−2^	1.00 × 10^−1^
				24010703	ATC provision of flight information	1.15 × 10^−2^	1.00 × 10^−1^
				25050000	ATM service personnel operating procedures/instructions	2.30 × 10^−2^	2.00 × 10^−1^
2020202	ANS clearance to wrong altitude	1.15 × 10^−2^	7.14 × 10^−2^	24010704	ATC provision of a minimum safe flight level/altitude/height/sector altitude	1.15 × 10^−2^	6.67 × 10^−2^
2020300	Communication between pilot and ANS	6.90 × 10^−2^	4.29 × 10^−1^	12251800	Pilot’s radiotelephony phraseology	1.15 × 10^−2^	5.56 × 10^−2^
				12252600	Pilot’s air/ground/air communication	4.60 × 10^−2^	2.22 × 10^−1^
				22080101	ATM’s internal coordination of civil sectors in the same unit	2.30 × 10^−2^	1.11 × 10^−1^
				24010101	ATC use of phraseology	1.15 × 10^−2^	5.56 × 10^−2^
				24010102	ATC use of readback/hearback error detection	4.60 × 10^−2^	2.22 × 10^−1^
				24010103	Blocked communication	2.30 × 10^−2^	1.11 × 10^−1^
				24010107	ATC requirement for the acknowledgement of information by the Pilot	1.15 × 10^−2^	5.56 × 10^−2^
				24010301	ATC requirement for the acknowledgement of information by the ATCO	1.15 × 10^−2^	5.56 × 10^−2^
2020508	Clearance deviation - approach	1.15 × 10^−2^	7.14 × 10^−2^	23020400	ATC use of clearance procedure	1.15 × 10^−2^	3.33 × 10^−1^
4010100	ANS operational communications	2.30 × 10^−2^	1.43 × 10^−1^	12252600	Pilot’s air/ground/air communication	1.15 × 10^−2^	6.67 × 10^−2^
				22080101	ATM’s internal coordination of civil sectors in the same unit	1.15 × 10^−2^	6.67 × 10^−2^
				24010102	ATC use of readback/hearback error detection	1.15 × 10^−2^	6.67 × 10^−2^
4010400	ANS conflict detection and resolution	1.38 × 10^−1^	8.57 × 10^−1^	22060100	ATM’s monitoring of A/C	3.45 × 10^−2^	5.26 × 10^−2^
				22080303	Revision of ATM’s coordination procedures	1.15 × 10^−2^	1.75 × 10^−2^
				22100600	Briefing for the hand-over/take-over	1.15 × 10^−2^	1.75 × 10^−2^
				22100700	Familiarization with traffic during the hand-over/take-over	1.15 × 10^−2^	1.75 × 10^−2^
				22120100	ATM’s strategic planning for conflict detection	5.75 × 10^−2^	8.77 × 10^−2^
				22120200	ATM’s tactical execution of the conflict detection strategy	6.90 × 10^−2^	1.05 × 10^−1^
				22130101	ATM’s horizontal conflict resolution by radar vectoring/monitoring	1.15 × 10^−2^	1.75 × 10^−2^
				23010300	Clearance procedure	3.45 × 10^−2^	5.26 × 10^−2^
				24010604	ATC provision of a short term conflict alert (STCA) warning	2.30 × 10^−2^	3.51 × 10^−2^
				27030000	ATC monitoring of sector traffic load	1.15 × 10^−2^	1.75 × 10^−2^
4010600	ANS handing over/taking over procedure	3.45 × 10^−2^	2.14 × 10^−1^	22080101	ATM’s internal coordination of civil sectors in the same unit	3.45 × 10^−2^	4.29 × 10^−1^
				23010300	Clearance procedure	1.15 × 10^−2^	1.43 × 10^−1^
4050300	Failure of surveillance	1.15 × 10^−2^	7.14 × 10^−2^	22060100	ATM’s monitoring of A/C	1.15 × 10^−2^	5.00 × 10^−1^
				24010705	ATC provision of delay related information	1.15 × 10^−2^	5.00 × 10^−1^
4070400	Air space capacity reduction	4.60 × 10^−2^	2.86 × 10^−1^	22080103	ATM’s internal coordination of military sectors in the same unit	1.15 × 10^−2^	1.25 × 10^−1^
				22100300	Airspace during the hand-over/take-over	1.15 × 10^−2^	1.25 × 10^−1^
				27030000	ATC monitoring of sector traffic load	2.30 × 10^−2^	2.50 × 10^−1^
				27050200	Factors relating coordination with ATFM	1.15 × 10^−2^	1.25 × 10^−1^
				41100300	Runway obstruction	1.15 × 10^−2^	1.25 × 10^−1^
				52020400	Tailwind	1.15 × 10^−2^	1.25 × 10^−1^
				52031400	Cloud amount restricting visibility	1.15 × 10^−2^	1.25 × 10^−1^

**Table A2:** CPT of Events and Descriptive Factors under Scenario of LOS in Commercial Aviation—Severity B.

Adverse Events (E)	Event Definition	P(E)	P(E|Severity)	Descriptive Factors (DF)	Descriptive Factor Definition	P(DF)	P(DF|E)
1230000	Communication systems	1.15 × 10^−2^	1.37 × 10^−2^	21010900	Headsets	1.15 × 10^−2^	5.00 × 10^−1^
				24010103	Blocked communication	1.15 × 10^−2^	5.00 × 10^−1^
2020201	ANS erroneous clearance	8.05 × 10^−2^	9.59 × 10^−2^	25050000	ATM service personnel operating procedures/instructions	5.75 × 10^−2^	5.00 × 10^−1^
				27030000	ATC monitoring of sector traffic load	2.30 × 10^−2^	2.00 × 10^−1^
				24010105	ATC call-sign confusion	1.15 × 10^−2^	1.00 × 10^−1^
				23020400	ATC use of clearance procedure	1.15 × 10^−2^	1.00 × 10^−1^
				12230900	Pilot’s operation of emergency brakes	1.15 × 10^−2^	1.00 × 10^−1^
				23020700	ATC use of descent procedure	1.15 × 10^−2^	1.00 × 10^−1^
				22060100	ATM’s monitoring of A/C	1.15 × 10^−2^	1.00 × 10^−1^
2020202	ANS clearance to wrong altitude	1.61 × 10^−1^	1.92 × 10^−1^	25050000	ATM service personnel operating procedures/instructions	1.15 × 10^−1^	6.67 × 10^−1^
				22120100	ATM’s strategic planning for conflict detection	1.15 × 10^−2^	6.67 × 10^−2^
				27030000	ATC monitoring of sector traffic load	2.30 × 10^−2^	1.33 × 10^−1^
				23020400	ATC use of clearance procedure	1.15 × 10^−2^	6.67 × 10^−2^
				12240600	The rate of descent of the aircraft	1.15 × 10^−2^	6.67 × 10^−2^
				22060100	ATM’s monitoring of A/C	5.75 × 10^−2^	3.33 × 10^−1^
				23020500	ATC use of climb procedure	1.15 × 10^−2^	6.67 × 10^−2^
				24010105	ATC call-sign confusion	1.15 × 10^−2^	6.67 × 10^−2^
2020300	Communication between pilot and ANS	1.38 × 10^−1^	1.64 × 10^−1^	12252600	Pilot’s air/ground/air communication	4.60 × 10^−2^	2.22 × 10^−1^
				24010102	ATC use of readback/hearback error detection	8.05 × 10^−2^	3.89 × 10^−1^
				12251800	Pilot’s radiotelephony phraseology	2.30 × 10^−2^	1.11 × 10^−1^
				24010101	ATC use of phraseology	4.60 × 10^−2^	2.22 × 10^−1^
				52031600	Thunderstorm	1.15 × 10^−2^	5.56 × 10^−2^
				12251400	Pilot’s action in respect to instruction	1.15 × 10^−2^	5.56 × 10^−2^
				24010105	ATC call-sign confusion	1.15 × 10^−2^	5.56 × 10^−2^
				22060200	ATM’s monitoring of frequencies	1.15 × 10^−2^	5.56 × 10^−2^
				25050000	ATM service personnel operating procedures/instructions	1.15 × 10^−2^	5.56 × 10^−2^
2020505	Clearance deviation - take-off	3.45 × 10^−2^	4.11 × 10^−2^	23020600	ATC use of departure procedure	1.15 × 10^−2^	3.33 × 10^−1^
				22100600	Briefing for the hand-over/take-over	1.15 × 10^−2^	3.33 × 10^−1^
				23020500	ATC use of climb procedure	1.15 × 10^−2^	3.33 × 10^−1^
				22050100	A/C performance differences	2.30 × 10^−2^	6.67 × 10^−1^
				23020400	ATC use of clearance procedure	1.15 × 10^−2^	3.33 × 10^−1^
2020506	Clearance deviation - en-route	6.90 × 10^−2^	8.22 × 10^−2^	23020700	ATC use of descent procedure	2.30 × 10^−2^	3.33 × 10^−1^
				24010703	ATC provision of flight information	1.15 × 10^−2^	1.67 × 10^−1^
				23020500	ATC use of climb procedure	3.45 × 10^−2^	5.00 × 10^−1^
				22090000	ATM’s traffic transfer	1.15 × 10^−2^	1.67 × 10^−1^
				12251800	Pilot’s radiotelephony phraseology	1.15 × 10^−2^	1.67 × 10^−1^
				22100600	Briefing for the hand-over/take-over	1.15 × 10^−2^	1.67 × 10^−1^
				52020400	Tailwind	1.15 × 10^−2^	1.67 × 10^−1^
				23010300	Clearance procedure	1.15 × 10^−2^	1.67 × 10^−1^
				23010200	AWY/Route approach procedure	1.15 × 10^−2^	1.67 × 10^−1^
2020508	Clearance deviation - approach	2.30 × 10^−2^	2.74 × 10^−2^	24010101	ATC use of phraseology	1.15 × 10^−2^	3.33 × 10^−1^
				12210900	Pilot’s obstacle clearance judgement	1.15 × 10^−2^	3.33 × 10^−1^
				12251400	Pilot’s action in respect to instruction	1.15 × 10^−2^	3.33 × 10^−1^
				52031400	Cloud amount restricting visibility	1.15 × 10^−2^	3.33 × 10^−1^
				22050100	A/C performance differences	1.15 × 10^−2^	3.33 × 10^−1^
2020509	Clearance deviation - holding	1.15 × 10^−2^	1.37 × 10^−2^	23020400	ATC use of clearance procedure	1.15 × 10^−2^	1.00 × 10^0^
2020513	Clearance deviation - special procedure	2.30 × 10^−2^	2.74 × 10^−2^	12251400	Pilot’s action in respect to instruction	2.30 × 10^−2^	1.00 × 10^0^
				24010102	ATC use of readback/hearback error detection	2.30 × 10^−2^	1.00 × 10^0^
2020517	Deviation from clearance - assigned flight level	1.03 × 10^−1^	1.23 × 10^−1^	12251500	Pilot’s action in respect to ATC clearance	8.05 × 10^−2^	7.78 × 10^−1^
				52020500	Crosswind	2.30 × 10^−2^	2.22 × 10^−1^
				22060100	ATM’s monitoring of A/C	1.15 × 10^−2^	1.11 × 10^−1^
				12232800	Pilot’s operation of communication equipment	1.15 × 10^−2^	1.11 × 10^−1^
				24010102	ATC use of readback/hearback error detection	1.15 × 10^−2^	1.11 × 10^−1^
				12251400	Pilot’s action in respect to instruction	1.15 × 10^−2^	1.11 × 10^−1^
				52031600	Thunderstorm	1.15 × 10^−2^	1.11 × 10^−1^
				11222000	Speed-attitude correction system	1.15 × 10^−2^	1.11 × 10^−1^
				12230900	Pilot’s operation of emergency brakes	1.15 × 10^−2^	1.11 × 10^−1^
				12252200	Pilot’s action in respect to standard operating procedure	1.15 × 10^−2^	1.11 × 10^−1^
				25050000	ATM service personnel operating procedures/instructions	1.15 × 10^−2^	1.11 × 10^−1^
2020519	Deviation from clearance - assigned or specified speed	2.30 × 10^−2^	2.74 × 10^−2^	12251500	Pilot’s action in respect to ATC clearance	1.15 × 10^−2^	5.00 × 10^−1^
				12240700	The flying speed of the aircraft	1.15 × 10^−2^	5.00 × 10^−1^
				12252600	Pilot’s air/ground/air communication	1.15 × 10^−2^	5.00 × 10^−1^
2020522	Deviation from clearance - climb/descent conditional clearance	1.15 × 10^−2^	1.37 × 10^−2^	24010101	ATC use of phraseology	1.15 × 10^−2^	1.00 × 10^0^
				12210500	Pilot’s perception of visual/oral warning	1.15 × 10^−2^	1.00 × 10^0^
2020805	Deviation from approach procedure	3.45 × 10^−2^	4.11 × 10^−2^	24010703	ATC provision of flight information	2.30 × 10^−2^	6.67 × 10^−1^
				23020300	ATC use of approach procedure	3.45 × 10^−2^	1.00 × 10^0^
				24010101	ATC use of phraseology	1.15 × 10^−2^	3.33 × 10^−1^
2100100	Diversion due to weather conditions	3.45 × 10^−2^	4.11 × 10^−2^	52031400	Cloud amount restricting visibility	1.15 × 10^−2^	3.33 × 10^−1^
				52010200	Instrument meteorological conditions	1.15 × 10^−2^	3.33 × 10^−1^
				52021200	Turbulence in cloud	1.15 × 10^−2^	3.33 × 10^−1^
2170200	Wrong runway selected	1.15 × 10^−2^	1.37 × 10^−2^	21040200	ATM’s information data system	1.15 × 10^−2^	1.00 × 10^0^
				24010304	Information input error in the ATC operations	1.15 × 10^−2^	1.00 × 10^0^
4010100	ANS operational communications	1.49 × 10^−1^	1.78 × 10^−1^	22080203	ATM’s coordination with an adjacent civil sector	6.90 × 10^−2^	4.00 × 10^−1^
				22090000	ATM’s traffic transfer	6.90 × 10^−2^	4.00 × 10^−1^
				22080101	ATM’s internal coordination of civil sectors in the same unit	2.30 × 10^−2^	1.33 × 10^−1^
				24010703	ATC provision of flight information	1.15 × 10^−2^	6.67 × 10^−2^
				23020700	ATC use of descent procedure	1.15 × 10^−2^	6.67 × 10^−2^
				22080103	ATM’s internal coordination of military sectors in the same unit	1.15 × 10^−2^	6.67 × 10^−2^
				22100600	Briefing for the hand-over/take-over	2.30 × 10^−2^	1.33 × 10^−1^
				22080201	ATM’s coordination with an adjacent civil unit	1.15 × 10^−2^	6.67 × 10^−2^
				25050000	ATM service personnel operating procedures/instructions	1.15 × 10^−2^	6.67 × 10^−2^
				24010106	ATC transfer of communication	1.15 × 10^−2^	6.67 × 10^−2^
4010200	ANS operational information provisions	2.30 × 10^−2^	2.74 × 10^−2^	24010703	ATC provision of flight information	2.30 × 10^−2^	1.00 × 10^0^
				27030000	ATC monitoring of sector traffic load	1.15 × 10^−2^	5.00 × 10^−1^
4010300	ANS separation provision	2.30 × 10^−2^	2.74 × 10^−2^	24010703	ATC provision of flight information	2.30 × 10^−2^	1.00 × 10^0^
				21020103	ATM’s use of the instrument landing system	1.15 × 10^−2^	5.00 × 10^−1^
				23020300	ATC use of approach procedure	1.15 × 10^−2^	5.00 × 10^−1^
				22060100	ATM’s monitoring of A/C	1.15 × 10^−2^	5.00 × 10^−1^
				23020700	ATC use of descent procedure	1.15 × 10^−2^	5.00 × 10^−1^
4010400	ANS conflict detection and resolution	5.17 × 10^−1^	6.16 × 10^−1^	23010201	Surveillance radar element of a precision approach radar system approach	1.15 × 10^−2^	1.75 × 10^−2^
				22130101	ATM’s horizontal conflict resolution by radar vectoring/monitoring	1.26 × 10^−1^	1.93 × 10^−1^
				24010703	ATC provision of flight information	4.60 × 10^−2^	7.02 × 10^−2^
				23010300	Clearance procedure	1.15 × 10^−2^	1.75 × 10^−2^
				22060100	ATM’s monitoring of A/C	5.75 × 10^−2^	8.77 × 10^−2^
				23020700	ATC use of descent procedure	8.05 × 10^−2^	1.23 × 10^−1^
				22110200	ATM’s updating of a flight plan	1.15 × 10^−2^	1.75 × 10^−2^
				23020600	ATC use of departure procedure	1.15 × 10^−2^	1.75 × 10^−2^
				23020400	ATC use of clearance procedure	4.60 × 10^−2^	7.02 × 10^−2^
				27060100	ATC assistance to the ATC in recovering control of traffic	2.30 × 10^−2^	3.51 × 10^−2^
				22120100	ATM’s strategic planning for conflict detection	3.45 × 10^−2^	5.26 × 10^−2^
				23020300	ATC use of approach procedure	2.30 × 10^−2^	3.51 × 10^−2^
				24010102	ATC use of readback/hearback error detection	2.30 × 10^−2^	3.51 × 10^−2^
				22120200	ATM’s tactical execution of the conflict detection strategy	2.30 × 10^−2^	3.51 × 10^−2^
				22130200	ATM’s vertical conflict resolution	8.05 × 10^−2^	1.23 × 10^−1^
				12252600	Pilot’s air/ground/air communication	1.15 × 10^−2^	1.75 × 10^−2^
				24010604	ATC provision of a short term conflict alert (STCA) warning	5.75 × 10^−2^	8.77 × 10^−2^
				52031600	Thunderstorm	1.15 × 10^−2^	1.75 × 10^−2^
				22090000	ATM’s traffic transfer	1.15 × 10^−2^	1.75 × 10^−2^
				22130300	ATM’s conflict resolution by planned controller action	1.15 × 10^−2^	1.75 × 10^−2^
				24010101	ATC use of phraseology	1.15 × 10^−2^	1.75 × 10^−2^
				27030000	ATC monitoring of sector traffic load	2.30 × 10^−2^	3.51 × 10^−2^
				24010605	ATC provision of airborne proximity warning	1.15 × 10^−2^	1.75 × 10^−2^
				12251500	Pilot’s action in respect to ATC clearance	1.15 × 10^−2^	1.75 × 10^−2^
				25050000	ATM service personnel operating procedures/instructions	3.45 × 10^−2^	5.26 × 10^−2^
				24010105	ATC call-sign confusion	1.15 × 10^−2^	1.75 × 10^−2^
4010500	ANS handling of accidents/incidents/emergency	1.15 × 10^−2^	1.37 × 10^−2^	23020800	ATC use of emergency procedure	1.15 × 10^−2^	1.00 × 10^0^
				22060100	ATM’s monitoring of A/C	1.15 × 10^−2^	1.00 × 10^0^
				26070000	ATM handling of A/C unusual/emergency situation	1.15 × 10^−2^	1.00 × 10^0^
				23010700	Emergency procedure	1.15 × 10^−2^	1.00 × 10^0^
4010600	ANS handing over/taking over procedure	4.60 × 10^−2^	5.48 × 10^−2^	22080203	ATM’s coordination with an adjacent civil sector	2.30 × 10^−2^	2.86 × 10^−1^
				25050000	ATM service personnel operating procedures/instructions	1.15 × 10^−2^	1.43 × 10^−1^
				27030000	ATC monitoring of sector traffic load	2.30 × 10^−2^	2.86 × 10^−1^
				22090000	ATM’s traffic transfer	1.15 × 10^−2^	1.43 × 10^−1^
				22080101	ATM’s internal coordination of civil sectors in the same unit	1.15 × 10^−2^	1.43 × 10^−1^
				22100600	Briefing for the hand-over/take-over	1.15 × 10^−2^	1.43 × 10^−1^
				27010300	ATC rostering/sector opening in relation to expected traffic	1.15 × 10^−2^	1.43 × 10^−1^
4050300	Failure of surveillance	1.15 × 10^−2^	1.37 × 10^−2^	21030401	ATM’s use of secondary area radar	1.15 × 10^−2^	5.00 × 10^−1^
4070400	Air space capacity reduction	4.60 × 10^−2^	5.48 × 10^−2^	52010200	Instrument meteorological conditions	1.15 × 10^−2^	1.25 × 10^−1^
				27030000	ATC monitoring of sector traffic load	3.45 × 10^−2^	3.75 × 10^−1^
				24010301	ATC requirement for the acknowledgement of information by the ATCO	1.15 × 10^−2^	1.25 × 10^−1^
				23021100	ATC use of holding procedure	1.15 × 10^−2^	1.25 × 10^−1^
				22100300	Airspace during the hand-over/take-over	1.15 × 10^−2^	1.25 × 10^−1^

## Appendix B. ADREP Taxonomy Code of Events and Descriptive Factors

**Table A3:** ADREP taxonomy code of events.

Event Code	Event Description
1230000	Communication systems
2020201	ANS erroneous clearance
2020202	ANS clearance to wrong altitude
2020300	Communication between pilot and ANS
2020505	Clearance deviation - take-off
2020506	Clearance deviation - en-route
2020508	Clearance deviation - approach
2020509	Clearance deviation - holding
2020513	Clearance deviation - special procedure
2020517	Deviation from clearance - assigned flight level
2020519	Deviation from clearance - assigned or specified speed
2020522	Deviation from clearance - climb/descent conditional clearance
2020805	Deviation from approach procedure
2100100	Diversion due to weather conditions
2170200	Wrong runway selected
4010100	ANS operational communications
4010200	ANS operational information provisions
4010300	ANS separation provision
4010400	ANS conflict detection and resolution
4010500	ANS handling of accidents/incidents/emergency
4010600	ANS handing over/taking over procedure
4050300	Failure of surveillance
4070400	Air space capacity reduction

**Table A4:** ADREP taxonomy code of descriptive factors.

Descriptive Factor Code	Descriptive Factor Description
11222000	Speed-attitude correction system
12210500	Pilot’s perception of visual/oral warning
12210900	Pilot’s obstacle clearance judgement
12230900	Pilot’s operation of emergency brakes
12232800	Pilot’s operation of communication equipment
12240600	The rate of descent of the aircraft
12240700	The flying speed of the aircraft
12251400	Pilot’s action in respect to instruction
12251500	Pilot’s action in respect to ATC clearance
12251800	Pilot’s radiotelephony phraseology
12252200	Pilot’s action in respect to standard operating procedure
12252600	Pilot’s air/ground/air communication
21010900	Headsets
21020103	ATM’s use of the instrument landing system
21030401	ATM’s use of secondary area radar
21040200	ATM’s information data system
22050100	A/C performance differences
22060100	ATM’s monitoring of A/C
22060200	ATM’s monitoring of frequencies
22080101	ATM’s internal coordination of civil sectors in the same unit
22080103	ATM’s internal coordination of military sectors in the same unit
22080201	ATM’s coordination with an adjacent civil unit
22080203	ATM’s coordination with an adjacent civil sector
22080303	Revision of ATM’s coordination procedures
22090000	ATM’s traffic transfer
22100300	Airspace during the hand-over/take-over
22100600	Briefing for the hand-over/take-over
22100700	Familiarization with traffic during the hand-over/take-over
22110200	ATM’s updating of a flight plan
22120100	ATM’s strategic planning for conflict detection
22120200	ATM’s tactical execution of the conflict detection strategy
22130101	ATM’s horizontal conflict resolution by radar vectoring/monitoring
22130200	ATM’s vertical conflict resolution
22130300	ATM’s conflict resolution by planned controller action
23010200	AWY/Route approach procedure
23010201	Surveillance radar element of a precision approach radar system approach
23010300	Clearance procedure
23010700	Emergency procedure
23020300	ATC use of approach procedure
23020400	ATC use of clearance procedure
23020500	ATC use of climb procedure
23020600	ATC use of departure procedure
23020700	ATC use of descent procedure
23020800	ATC use of emergency procedure
23021100	ATC use of holding procedure
24010101	ATC use of phraseology
24010102	ATC use of readback/hearback error detection
24010103	Blocked communication
24010105	ATC call-sign confusion
24010106	ATC transfer of communication
24010107	ATC requirement for the acknowledgement of information by the Pilot
24010301	ATC requirement for the acknowledgement of information by the ATCO
24010304	Information input error in the ATC operations
24010604	ATC provision of a short term conflict alert (STCA) warning
24010605	ATC provision of airborne proximity warning
24010703	ATC provision of flight information
24010704	ATC provision of a minimum safe flight level/altitude/height/sector altitude
24010705	ATC provision of delay related information
25050000	ATM service personnel operating procedures/instructions
26070000	ATM handling of A/C unusual/emergency situation
27010300	ATC rostering/sector opening in relation to expected traffic
27030000	ATC monitoring of sector traffic load
27050200	Factors relating coordination with ATFM
27060100	ATC assistance to the ATC in recovering control of traffic
41100300	Runway obstruction
52010200	Instrument meteorological conditions
52020400	Tailwind
52020500	Crosswind
52021200	Turbulence in cloud
52031400	Cloud amount restricting visibility
52031600	Thunderstorm

## Appendix C. Groups of Descriptive Factors Associated to the Result of Mutual Information I_A∩B

**Table A5:** Associated DFs of severity B with I_A∩B = 0.

DF ID	DF Belonging to Severity B
11222000	Speed-attitude correction system
12210500	Pilot’s perception of visual/oral warning
12210900	Pilot’s obstacle clearance judgement
12230900	Pilot’s operation of emergency brakes
12240600	The rate of descent of the aircraft
12240700	The flying speed of the aircraft
12251400	Pilot’s action in respect to instruction
12251500	Pilot’s action in respect to ATC clearance
12252200	Pilot’s action in respect to standard operating procedure
21010900	Headsets
21020103	ATM’s use of the instrument landing system
21030401	ATM’s use of secondary area radar
21040200	ATM’s information data system
22050100	A/C performance differences
22060200	ATM’s monitoring of frequencies
22080201	ATM’s coordination with an adjacent civil unit
22080203	ATM’s coordination with an adjacent civil sector
22090000	ATM’s traffic transfer
22110200	ATM’s updating of a flight plan
22130200	ATM’s vertical conflict resolution
22130300	ATM’s conflict resolution by planned controller action
23010200	AWY/Route approach procedure
23010201	Surveillance radar element of a precision approach radar system approach
23010700	Emergency procedure
23020300	ATC use of approach procedure
23020500	ATC use of climb procedure
23020600	ATC use of departure procedure
23020700	ATC use of descent procedure
23020800	ATC use of emergency procedure
23021100	ATC use of holding procedure
24010105	ATC call-sign confusion
24010106	ATC transfer of communication
24010304	Information input error in the ATC operations
24010605	ATC provision of airborne proximity warning
26070000	ATM handling of A/C unusual/emergency situation
27010300	ATC rostering/sector opening in relation to expected traffic
27060100	ATC assistance to the ATC in recovering control of traffic
52010200	Instrument meteorological conditions
52020500	Crosswind
52021200	Turbulence in cloud
52031600	Thunderstorm

**Table A6:** Associated DFs of severity A with I_A∩B = 0.

DF ID	DF due to the Boundary Conditions
22080303	Revision of ATM’s coordination procedures
22100700	Familiarization with traffic during the hand-over/take-over
24010107	ATC requirement for the acknowledgement of information by the Pilot
24010704	ATC provision of a minimum safe flight level/altitude/height/sector altitude
24010705	ATC provision of delay related information
27050200	Factors relating coordination with ATFM
41100300	Runway obstruction

**Table A7:** Associated DFs of severity A and B with I_AB → 0.

DF ID	DF Belonging to Severity A or B Separately
12232800	Pilot’s operation of communication equipment
22080103	ATM’s internal coordination of military sectors in the same unit
22100600	Briefing for the hand-over/take-over
23020400	ATC use of clearance procedure
24010103	Blocked communication
24010301	ATC requirement for the acknowledgement of information by the ATCO
24010703	ATC provision of flight information
52020400	Tailwind
52031400	Cloud amount restricting visibility

**Table A8:** Associated DFs of severity A and B with I > 0.

DF ID	DF Shared in Severity A and B
12251800	Pilot’s radiotelephony phraseology
12252600	Pilot’s air/ground/air communication
22060100	ATM’s monitoring of A/C
22080101	ATM’s internal coordination of civil sectors in the same unit
22100300	Airspace during the hand-over/take-over
22120100	ATM’s strategic planning for conflict detection
22120200	ATM’s tactical execution of the conflict detection strategy
22130101	ATM’s horizontal conflict resolution by radar vectoring/monitoring
23010300	Clearance procedure
24010101	ATC use of phraseology
24010102	ATC use of readback/hearback error detection
24010604	ATC provision of a short term conflict alert (STCA) warning
25050000	ATM service personnel operating procedures/instructions
27030000	ATC monitoring of sector traffic load

## References

[1] Reason J. (1990). Human Error.

[2] Heinrich H.W. (1931). Industrial Accident Prevention: A Scientific Approach.

[3] Heinrich H.W., Roos N.R., Petersen D.C. (1980). Industrial Accident Prevention: A Safety Management Approach.

[4] Johnson A. Examining the foundation. Proceedings of the National Safety Council Congress & Expo.

[5] Sultana S., Andersen B.S., Haugen S. (2019). Identifying safety indicators for safety performance measurement using a system engineering approach. Process Saf. Environ. Prot..

[6] Kyriakidis M., Hirsch R., Majumdar A. (2012). Metro railway safety: An analysis of accident precursors. Saf. Sci..

[7] Golovina O., Perschewski M., Teizer J., König M. (2019). Algorithm for quantitative analysis of close call events and personalized feedback in construction safety. Autom. Constr..

[8] Marshall P., Hirmas A., Singer M. (2018). Heinrich’s pyramid and occupational safety: A statistical validation methodology. Saf. Sci..

[9] Prem K.P., Ng D., Mannan M.S. (2010). Harnessing database resources for understanding the profile of chemical process industry incidents. J. Loss Prev. Process Ind..

[10] Walker G. (2017). Redefining the incidents to learn from: Safety science insights acquired on the journey from black boxes to Flight Data Monitoring. Saf. Sci..

[11] Majumdar A., Dupuy M.D., Ochieng W.Y., Nalder P. (2006). Developing Safety Indicators for New Zealand Airspace: Analysis of Loss-of-Separation Incidents. Transp. Res. Rec. J. Transp. Res. Board.

[12] Nazeri Z., Donohue G., Sherry L. Analyzing Relationships Between Aircraft Accidents and Incidents. Proceedings of the International Conference on Research in Air Transportation (ICRAT 2008).

[13] Arnaldo Valdés R.M., Liang Cheng S.Z., Gómez Comendador V.F., Sáez Nieto F.J. (2018). Application of Bayesian Networks and Information Theory to Estimate the Occurrence of Mid-Air Collisions Based on Accident Precursors. Entropy.

[14] Liang Cheng S.Z., Arnaldo Valdés R.M., Gómez Comendador V.F., Román Cordón R. Analysis of accident precursor data for Mid Air Collision occurrences using expert build Bayesian Network model and Information Theory. Proceedings of the 8th European Conference for Aeronautics and Space Sciences (Eucass).

[15] Kim N.K., Jeon K.M., Kim H.K. (2019). Convolutional Recurrent Neural Network-Based Event Detection in Tunnels Using Multiple Microphones. Sensors.

[16] Nan K., Liu S., Du J., Liu H. (2019). Deep model compression for mobile platforms: A survey. Tsinghua Sci. Technol..

[17] ICAO (2016). International Standards and Recommended Practices Annex 11 to the Convention on International Civil Aviation Air Traffic Services.

[18] European Union (2014). Regulation (EU) No 376/2014 of the European Parliament and of the Council of 3 April 2014.

[19] Ceanita Informes Definitivos. https://www.seguridadaerea.gob.es/lang_castellano/g_r_seguridad/ceanita/informes_definitivos/default.aspx.

[20] Licu T., Cioran F., Hayward B., Lowe A. (2007). EUROCONTROL—Systemic Occurrence Analysis Methodology (SOAM)—A ‘Reason’-based organisational methodology for analysing incidents and accidents. Reliab. Eng. Syst. Saf..

[21] Eurocontrol (2005). EAM 2/GUI 8—Guidelines on the Systemic Occurrence Analysis Methodology (SOAM).

[22] Liang Cheng S.Z., Arnaldo Valdés R.M., Gómez Comendador V.F., Sáez Nieto F.J. (2019). A Case Study of Fishbone Sequential Diagram Application and ADREP Taxonomy Codification in Conventional ATM Incident Investigation. Symmetry.

[23] Ferrante O., Jouniaux P., Loo T., Nicolas G., Cabon P., Mollard R. (2004). Application of ADREP 2000 taxonomy for the analysis and the encoding of aviation accidents and incidents: A human factors approach. Hum. Factors Aerosp. Saf..

[24] Nadkarni S., Shenoy P.P. (2004). A causal mapping approach to constructing Bayesian networks. Decis. Support Syst..

[25] Damelin S.B., Miller W. (2011). The Mathematics of Signal Processing.

[26] Hu X., Zhang H., Ma D., Wang R. (2019). Status detection from spatial-temporal data in pipeline network using data transformation convolutional neural network. Neurocomputing.

[27] Nguyen H.D., McLachlan G. (2019). On approximations via convolution-defined mixture models. Commun. Stat. Theory Methods.

[28] Huang W., Lai K.K., Zhang J., Bao Y. Foreign Exchange Rates Forecasting with Multilayer Perceptrons Neural Network by Bayesian Learning. Proceedings of the 2008 Fourth International Conference on Natural Computation.

[29] Abdulhai B., Ritchie S.G. (1999). Enhancing the universality and transferability of freeway incident detection using a Bayesian-based neural network. Transp. Res. Part C Emerg. Technol..

[30] Gupta P., Schumann J. A tool for verification and validation of neural network based adaptive controllers for high assurance systems. Proceedings of the Eighth IEEE International Symposium on High Assurance Systems Engineering.

